# Older Adults’ Experiences With Using Wearable Devices: Qualitative Systematic Review and Meta-synthesis

**DOI:** 10.2196/23832

**Published:** 2021-06-03

**Authors:** Kevin Moore, Emma O'Shea, Lorna Kenny, John Barton, Salvatore Tedesco, Marco Sica, Colum Crowe, Antti Alamäki, Joan Condell, Anna Nordström, Suzanne Timmons

**Affiliations:** 1 Centre for Gerontology and Rehabilitation University College Cork Cork Ireland; 2 Tyndall Institute Cork Ireland; 3 Research, Development and Innovation Activities & Physiotherapy Education Karelia University of Applied Sciences Karelia Finland; 4 School of Computing, Engineering and Intelligent Systems Faculty of Computing, Engineering and the Built Environment Ulster University Coleraine United Kingdom; 5 Department of Public Health and Clinical Medicine Umeå University Umeå Sweden; 6 School of Sport Sciences The Arctic University of Norway Tromsø Norway

**Keywords:** wearable device, older adult, digital health, meta-synthesis, qualitative review, acceptance, adherence, mobile phone

## Abstract

**Background:**

Older adults may use wearable devices for various reasons, ranging from monitoring clinically relevant health metrics or detecting falls to monitoring physical activity. Little is known about how this population engages with wearable devices, and no qualitative synthesis exists to describe their shared experiences with long-term use.

**Objective:**

This study aims to synthesize qualitative studies of user experience after a multi-day trial with a wearable device to understand user experience and the factors that contribute to the acceptance and use of wearable devices.

**Methods:**

We conducted a systematic search in CINAHL, APA PsycINFO, PubMed, and Embase (2015-2020; English) with fixed search terms relating to *older adults* and *wearable devices*. A meta-synthesis methodology was used. We extracted themes from primary studies, identified key concepts, and applied reciprocal and refutational translation techniques; findings were synthesized into third-order interpretations, and finally, a “line-of-argument” was developed. Our overall goal was theory development, higher-level abstraction, and generalizability for making this group of qualitative findings more accessible.

**Results:**

In total, we reviewed 20 papers; 2 evaluated fall detection devices, 1 tested an ankle-worn step counter, and the remaining 17 tested activity trackers. The duration of wearing ranged from 3 days to 24 months. The views of 349 participants (age: range 51-94 years) were synthesized. Four key concepts were identified and outlined: motivation for device use, user characteristics (openness to engage and functional ability), integration into daily life, and device features. Motivation for device use is intrinsic and extrinsic, encompassing many aspects of the user experience, and appears to be as, if not more, important than the actual device features. To overcome usability barriers, an older adult must be motivated by the useful purpose of the device. A device that serves its intended purpose adds value to the user’s life. The user’s needs and the support structure around the device—aspects that are often overlooked—seem to play a crucial role in long-term adoption. Our “line-of-argument” model describes how motivation, ease of use, and device purpose determine whether a device is perceived to add value to the user’s life, which subsequently predicts whether the device will be integrated into the user’s life.

**Conclusions:**

The added value of a wearable device is the resulting balance of motivators (or lack thereof), device features (and their accuracy), ease of use, device purpose, and user experience. The added value contributes to the successful integration of the device into the daily life of the user. Useful device features alone do not lead to continued use. A support structure should be placed around the user to foster motivation, encourage peer engagement, and adapt to the user’s preferences.

## Introduction

### Background

Wearable health monitoring devices have seen a rapid rise in capability and popularity over the last two decades. These small wireless devices can monitor movements, improve physical activity, and facilitate ageing-in-place. Wearable devices temporarily and noninvasively attach to a person without hindering their movement and are often intended to be worn continuously. Examples include activity trackers (eg, Fitbit and smartwatches), fall detection devices, electromyography patches, and smart clothing.

Although older adults are not core consumers of wearable devices, their use of digital health technologies is increasing [1] in tandem with the expanding technological capabilities of wearable devices. Wearable devices can support “active ageing,” the process of enhancing quality of life as people age [2]. Technology creates an enabling environment that restores function and expands the participation of older adults in their health. Remote monitoring using wearable devices can aid independence and encourage older adults to manage stable chronic conditions by themselves. Clinicians can track patients’ health status remotely and communicate via video-based consultations [3]. Current wearable devices possess the ability to monitor a number of health metrics, including heart rate, blood oxygen levels, body temperature, physical activity, sleep, and blood pressure [4]. The older adult population is vulnerable to changes in their health conditions and may be burdened by frequent clinical visits. Wearable devices are well suited for monitoring older adults because they convey up-to-date health information and track health metrics over time. Wearable devices are intended to be worn continuously. For example, fall detection devices are worn all day, as falls occur unexpectedly. As these devices are used frequently, it is important to understand the barriers to acceptance and adherence. Factors such as trust, functionality, added value, ease of use, cost, stigma, and fear of dependence are examples of barriers to adoption [5].

Researchers have used a variety of methods to collect information from older adults regarding the acceptability of wearable devices: surveys [6,7], wear time [1], diaries [8,9], interviews [9], and focus groups [10,11]. Some studies collected information about general preferences regarding device design [11]; others allowed participants to interact with several devices before asking about preferred design features [12], in which participants used a wearable device for multiple days and then provided feedback [13-15].

### Objectives

Qualitative research methods are well suited to examine the user experience and may offer explanations for unexpected or anomalous findings in quantitative data [16] or uncover usability barriers that quantitative approaches often miss. Systematic reviews that combine the findings of multiple qualitative studies can identify common factors among studies and generalize their findings. No qualitative systematic review exists on older adults’ experiences of using any form of wearable device. Although each user experience is unique, a synthesis of studies may lead to a richer understanding of the integration of devices into the lives of users. Our objective is to better understand these experiences to inform future research efforts and to inspire device design to ensure a successful user experience.

We aim to apply a qualitative meta-synthesis process to the available qualitative data on older adults’ experiences with using wearable devices. Meta-synthesis is a form of interpretive synthesis that can be used in the review and evaluation of qualitative research studies. Our meta-synthesis is based on the principles of meta-ethnography [17], a method designed by Noblit and Hare [17] to synthesize ethnographic studies. A meta-synthesis differs from traditional meta-ethnography in that it allows for a variety of data analysis techniques besides ethnography (eg, phenomenology and grounded theory) to be synthesized together. It is an inductive method that compares, translates, and integrates concepts across studies, while also preserving the context of the primary data. This meta-synthesis is more in-depth than previous systematic reviews on wearable devices that summarized a group of studies [4,5,18-23], analyzed a series of trials [24], or reviewed the state of the art [25,26]. Our overall goal is theory development, higher-level abstraction, and generalizability for making this group of qualitative findings more accessible [27].

Our overarching research question is “What is the experience of older adults who took part in multi-day wearable device trials and what factors contribute to acceptance and use?”

## Methods

### Search Strategy

The inclusion and exclusion criteria (Textbox 1) were designed to accommodate various study designs as long as they contained the following core structure: an older person using a wearable device for multiple days and the subsequent qualitative analysis of that participant’s experience in relation to the wearable device. We created an inclusive search strategy to locate studies that used unusual jargons or unconventional study designs (Textbox 2). We also reviewed the search terms used in other systematic reviews in this area, consulted with colleagues in clinical and technical expertise in the area, and piloted various combinations of search terms to assess the sensitivity of the terms. KM searched four databases—CINAHL, APA PsycINFO, PubMed, and Embase—from January 2015 to January 2020 for studies published in English. Our date range intentionally excluded older wearable devices to minimize the differences between the capabilities of the devices used in the studies. Insights from before this period were recorded in a systematic review by Bergmann and McGregor [28]. This search was supplemented by searching Google Scholar, forward and backward searches of citation lists, and the publication lists of prominent researchers in the field.

**Inclusion and exclusion criteria guiding study selection.**

**Inclusion criteria**

- Peer-reviewed studies (in English)
- Published between January 2015 and January 2020
- Experiences of older adults using wearable devices
- Using a defined qualitative approach
- Presenting distinct qualitative data and results
- Qualitative data collected after the multi-day trial

**Exclusion criteria**

- Studies not in English or outside the time frame
- Not focused on older populations
- No primary qualitative data presented
- No continuous, multi-day trial component

**Search terms used in the search strings.**

**Search terms**

- (“wearable technology” OR “wearable sensor” OR “inertia sensor” OR “wireless sensor” OR accelerometer OR “Micro-Electrical-Mechanical-System” OR Actigraphy OR “inertial measurement unit” OR “motion monitor” OR “movement sensor” OR “wearable interface*” OR “body worn” OR wearable OR “wireless monitoring system” OR “activity tracker*” OR “activity-tracking” OR “activity sensor*” OR “activity assessment*” OR “fall detection” OR “wireless sensor networks”) AND (“user preference*” OR “user experience*” OR “user needs” OR preference* OR “patient centered” OR qualitative OR “focus group” OR perception* OR understanding OR acceptance OR adoption OR usability OR perspective) AND (“older adults” OR older OR ageing OR Parkinson’s OR Alzheimer’s OR Dementia OR stroke OR chronic) NOT (invasive OR implant*)

### Data Collection

**Figure 1:**
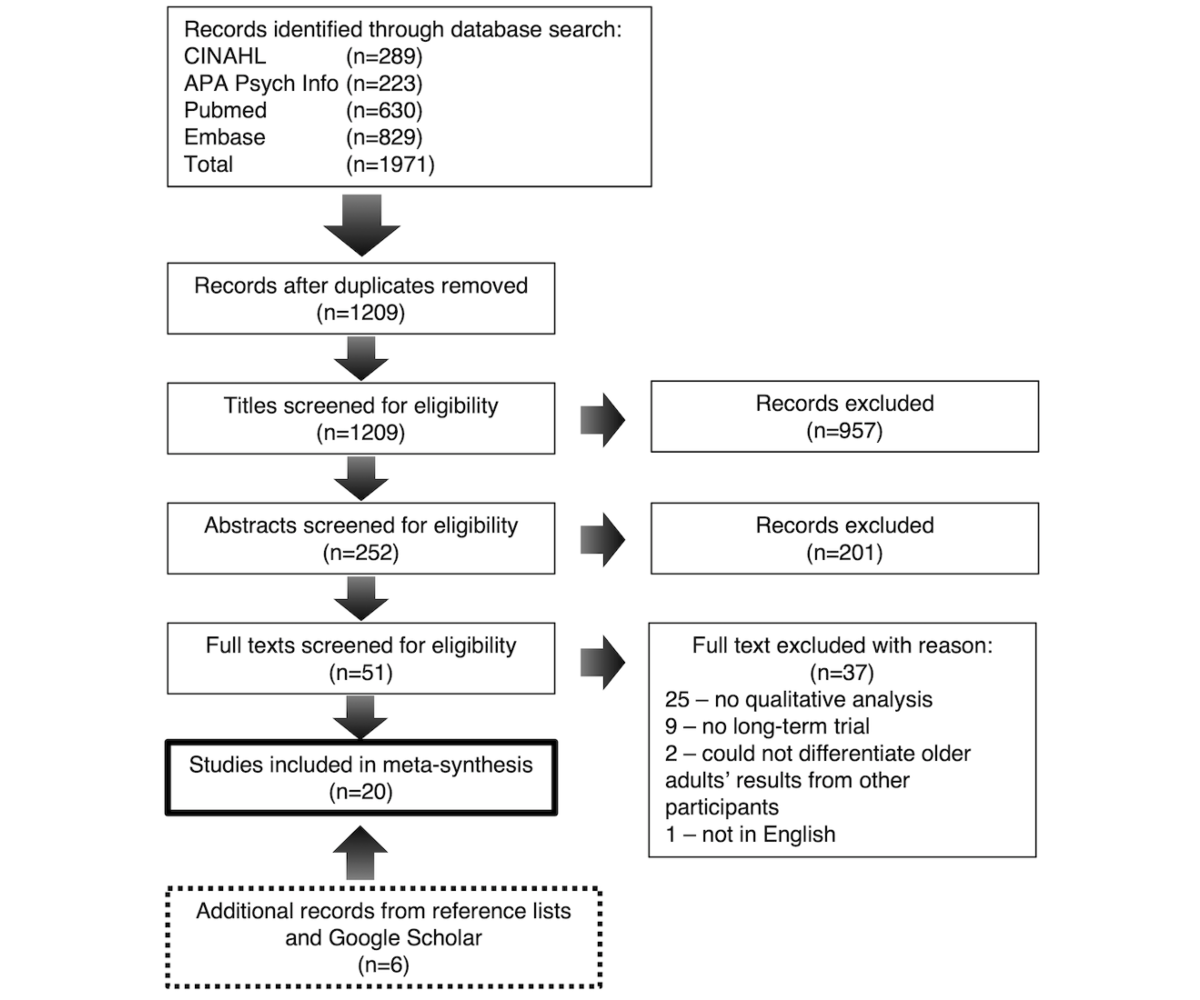
Preferred Reporting Items for Systematic Reviews and Meta-Analyses flowchart of study selection process.

KM exported the search results, removed duplicates, screened all titles and abstracts for inclusion, and reviewed eligible full-text articles against the inclusion or exclusion criteria (Textbox 1). LK screened a random sample of 200 abstracts (and full texts where the abstract indicated potential for inclusion) and confirmed the consistent application of the inclusion and exclusion criteria. A senior researcher, ST, provided guidance when eligibility based on a full-text review was unclear. Reasons for exclusion were recorded for all excluded studies (Figure 1).

### Data Extraction and Quality Appraisal

Data items extracted included information about the publication (date, authors, and study aims), study process (design, methods, and analysis), participant characteristics, device types and features, trial length, and relevant primary qualitative data (themes and quotations).

Although not required in a meta-ethnography, we assessed study quality to facilitate the critical reading of each study to gauge its potential contribution to the analysis (see Table S2 in Multimedia Appendix 1 [9,13-15,29-44] for the checklist). We used the Evaluation Tool for Qualitative Studies (ETQS) [45], as this provides detailed instructions on applying the evaluation criteria, unlike the CASP (Critical Appraisals Skills Programme) tool [46,47]. The ETQS guides the appraisal of the phenomenon studied and context issues; ethics; data collection, analysis, and researcher bias; and policy and practice implications [45]. Two authors (KM and LK) independently conducted the quality assessment. No studies were excluded based on ETQS results, as this often reflects the level of reporting transparency, rather than the actual research processes used [16,48].

### Data Analysis and Synthesis

The analysis (Textbox 3) was guided by a meta-ethnographic approach [17,48,49]. Initially, papers were read and reread to familiarize researchers with the study context, design, and findings. Individual themes were extracted and recorded using separate index cards. As the studies were methodologically heterogeneous, we preserved the authors’ original themes (and wording), where possible. Where appropriate, we extracted additional themes from the “discussion” and “conclusion” sections. Where studies were highly descriptive or simply listed participants’ quotes, we coded the “results” and “discussion” sections and generated themes from the presented data, noting that specific quotes, without the full conversation context, were challenging to code. Studies that presented minimal or overly descriptive results were mainly used to support or refute the themes identified in high-quality studies.

**Key steps involved in the synthesis and adapted from Noblit and Hare.**

**Key Steps**

- Identifying knowledge gaps and the literature available for a synthesis and developing research questions
- Defining the focus of the synthesis, locating relevant studies, and assessing the quality of the included studies
- Active reading of the studies to understand context and to extract relevant data
- Themes and concepts were identified in the “results” and “discussion” sections; authors’ interpretations were retained where possible; descriptive studies (without author-generated themes) were coded; and each theme was transferred to an index card along with contextual information, a narrative summary, and device characteristics.
- Index cards (each containing an extracted theme) were juxtaposed and grouped into general categories, categories were refined and subcategories emerged, and key concepts were identified.
- Returning to each study and comparing with the generated categories, using the context provided by the authors to re-evaluate the category placement of each index card, generating new index cards when the existing index cards do not represent the totality of the study results, and generating new subcategories and condensing others to better describe the results of the studies
- Compiling the participant raw data, index cards, and categories to produce overarching concepts that describe the results of the translation process
- Development of a line-of-argument synthesis and conceptual model

The themed index cards were physically juxtaposed and grouped into categories based on patterns of meaning, as related to the research question. Categories and subcategories were refined iteratively through constant comparison within and across studies. Each category was compared against each original study using (1) reciprocal translation (recognizing reoccurring themes or concepts across studies) and (2) refutational translation (recognizing dissimilar themes or concepts across studies, not explained by contextual factors). When all the data were collated and interpreted, several key concepts were defined (third order) and synthesized to develop an integrative “line-of-argument.”

We tracked the preferred and disliked device features throughout the analysis process. Where relevant, we used specific device features to support the key concepts. We summarized the preferred and disliked features, but no frequency analysis was performed because each study used different devices and not all studies included participant feedback on device features.

We reported our results in line with the eMERGe guidance, which has been described for use by researchers conducting meta-ethnography [50]. The search strategy results are presented in a Preferred Reporting Items for Systematic Reviews and Meta-analyses flow diagram (Figure 1).

## Results

### Overview

The database search returned 1971 results (Figure 1). After title or abstract screening and duplicate removal, 51 full-text records were reviewed, and 14 were considered eligible for inclusion. Backward or forward searching uncovered 6 additional eligible records to reach a total of 20 records for the synthesis.

### Characteristics of Included Studies

Of the 20 included records, 2 evaluated fall detection devices [14,29], 1 tested an ankle-worn step counter [30], and the remaining 17 examined wrist-worn activity trackers. The duration of use ranged from 3 days to 24 months. In some studies, users completed multiple trials with different devices [9,31-33]. In others, participants were randomly assigned to one of several devices [34,35]. The views of 349 participants (age: range 51-94 years) were synthesized, including those with previous breast cancer [32], obesity [36,37], resolving heart failure [30,37], Parkinson disease [38], dementia [15,39], and walking aids [9,29] and those who were fully independent and healthy [9,13,14,29,31-35,37,40-44]. Table 1 summarizes the results of the data extraction process.

**Table 1:** Articles included in the meta-synthesis and quality appraisal scores using the Evaluation Tool for Qualitative Studies.

Study	Method	Participants	Device	Trial duration	ETQS^a^ [45]
Abouzahra and Ghasemaghaei [13]	Interview pre- and posttrial^b^ Device data	44 participants Aged 65-75 years	Fitbit: AT^c^+SP^d^—wrist-worn	1 week	6
Batsis [36]	Surveys Interview^b^	8 participants Aged 65-80 years Rural; obese	Fitbit: PD^e^+SP—waist clip	4 weeks	5
Demiris et al [14]	Interview (×2)^b^ Fall or device log	18 participants Aged ≥62 years Slight fall risk	FDD^f^—clip or lanyard	4 months	8
Ehn et al [9]	Interview^b^ Follow-up meeting Activity diary	8 participants Aged 75-90 years	Withings Activité Pop: AT+SP—wrist-worn Jawbone UP3: AT+SP—wrist-worn	9-10 days for each device	10
Farina et al [15]	Device diary Questionnaire Dyadic interview^b^	26 participants Aged 65-90 years Alzheimer and dementia	GENEactiv Original: AT—wrist-worn	1 month	8
Fausset et al [34]	Questionnaire—interviews pre- or posttrial^g^ Daily diary	8 participants Aged 61-69 years	Striiv: PD—clip Fitbit: PD—clip Nike+FuelBand: AT—wrist-worn MyFitnessPal: web-based	Randomly assigned one device for 2 weeks	5
Floegel et al [30]	Interview^b^	27 participants Aged 62-90 years Heart failure requiring hospitalization	Tractivity: AT+SP—ankle-worn	1 month	6
Hermanns et al [38]	Surveys^g^ Interview^b^	5 participants Aged 65-81 years Stage I-IV Parkinson disease	Fitbit: AT+tablet—wrist-worn	12 weeks	8
Kononova et al [37]	Focus group^a^	48 (nonusers, short-term, former, and long-term users) Aged 65-94 years	Garmin Vivofit 2: AT—wrist-worn	2-4 weeks	9
Lee et al [40]	Adoption and usability surveys Biweekly interviews^b^	17 participants Aged 65-85 years	Nokia Go: AT—wrist-worn	14 weeks	8
Mercer et al [31]	Questionnaire Focus groups^b^	32 participants Aged 52-84 years	Fitbit Zip: PD—clip Jawbone Up 24: AT—wrist-worn Misfit Shine: AT—wrist-worn or clip Withings Pulse: AT—wrist-worn PD—clip	5 devices, each for ≥3 days (≥15 days total)	8
Nguyen et al [32]	Focus groups^b^	14 participants Aged 51-64 years	Fitbit One: PD—clip Jawbone Up 24: AT—wrist-worn Garmin Vivofit 2: AT—wrist-worn Garmin Vivosmart: AT—wrist-worn Garmin Vivoactive: AT—wrist-worn Polar A300: AT—wrist-worn	Assigned 2 devices; 2 weeks per device, 4 weeks total	8
Preusse et al [35]	Questionnaire; interview^h^	16 participants Aged 65-73 years	MyFitnessPal: web-based Fitbit One: AT—wrist-worn+web-based	28 days	7
Puri et al [33]	Questionnaire Interview with sample^i^	20 participants Aged 55-84 years	Microsoft Band: AT—wrist-worn Mi Band: AT—wrist-worn	Each device for 3 weeks, 6 weeks total	9
Rosales et al [41]	Interviews (×2)^h^	5 participants Aged ≥65 years Smartphone users	Moto G 360: SW—wrist-worn	12-24 months	6
Schlomann et al [43]	Group discussion^b^	6 participants Aged 67-78 years	AT+SP—wrist-worn	1 month	6
Schlomann [42]	Interviews (×2)^b,h^	6 participants Aged 60-78 years Smartphone users	ViFit: AT—wrist-worn	1 year	6
Thilo et al [29]	Daily diary Focus group^h^	15 participants Aged 75-92 years History of falls	FDD—torso patch	9 days	8
Thorpe et al [39]	Interview^h^ Sensor data	6 participants Aged 65-78 years Dementia	Sony SmartWatch 3: SW+SP—wrist-worn	9 weeks	6
Zhou et al [44]	Questionnaire Interview^b^	20 participants Aged 58-68 years	37 Degree Technology: AT+SP—wrist-worn	3 months	6

^a^ETQS: Evaluation Tool for Qualitative Studies; maximum score is 10.

^b^Inductive analysis.

^c^AT: activity tracker.

^d^SP: smartphone.

^e^PD: pedometer.

^f^FDD: fall detection device.

^g^Descriptive analysis.

^h^Deductive analysis.

^i^Directed content analysis.

### Translation

This study found four key concepts, comprising 12 subthemes that characterize the collective experience of trial participants (Textbox 4).

**Results of the reciprocal and refutation translation process.**

**Category 1: Openness to engage and functional ability of the user**

- Age-related physiology and comorbidities [14,32]
  Physical limitations such as hand dexterity
  Slower processing speeds in time of need
  Inactive lifestyle does not warrant activity tracker
- Sense of independence [14,15,29,33]
  Confidence in abilities to remember procedures
  Change in routine (battery life; attached to phone)
  Subjective norm, not burden on family
  Access to instructions and training
- Exploration and use of device features [9,32,43]
  Interest in diverse features and uses
  Confidence to explore means maximized benefits
  Technology experience means ability to troubleshoot
  Instructions to overcome hurdles
- Self-efficacy for technology [9,13,31]
  Skill to control and manipulate technology
  Perception of one’s own ability to use technology
  Insecurities of using the system reduced usage

**Category 2: Motivation for device use**

- Awareness of physical activity levels [9,31,32,37,38]
  Real versus perceived activity levels
  Awareness is not the same as motivation
  Awareness is a catalyst, not a creator of motivation
- Internal influences [9,13,37,39]
  Intrinsic motivation required for behavior change
  Achieving personal goals is satisfying
  Desire to improve health and fitness
  Expectation-confirmation theory; if the device meets the user’s expectation, they may be more likely to adopt the device
- Quantification and feedback [9,30,33,36,37,39,40,42-44]
  Personalized goals and feedback can motivate
  Data visualization helps to plan and monitor goals
  Health data visualization connects user to the purpose of the device
  Poor or absent feedback diminishes value
- Emotions invoked by the device [9,36]
  Connected to feedback
  Relationship with the device
  Negative feelings toward the device can lead to abandonment
  Emotional attachment to an external motivator can be a positive driving force
- Social capital and encouragement [13,15,36,39,41,44]
  Social capital promotes continued use
  Wearables as adjunct to social support
  Peer support, interaction, and communication
  Help with troubleshooting
  An external influence; boost motivation
- Promotion by health care staff [31,32]
  Benefits of involvement by health sector
  Motivated to use if part of the treatment plan
  Input from care team to overcome barriers and meet goals

**Category 3: Integration into daily life [9,13-15,34-37,39,41-43]**

- Ease of integration is determined by features, day-to-day function, purpose, and reliability of the device
- Cumbersome or annoying design features hinder integration
- Lack of desired features diminishes value of the device
- The device cannot serve its function if it is unreliable and difficult to use
- Reliability issues affect routine and can lead to stigma and embarrassment
- Device issues reduce motivation and diminish the value of devices

**Category 4: Device features [9,13,14,29,30,32,33,35,37,40,43]**

- Preferred features (in no particular order): waterproof, step count, easy-to-read display format, GPS (security in case of a fall or getting lost), looks like a watch, comfortable location on the body (generally wrist or ankle), secure attachment, smaller, long battery life, fewer notifications, does not interfere with clothing, personalized notifications or alarms, thin and flexible band, simple attachment (easy to use with limited dexterity), comfortable to wear at night, easy to work (intelligibility), more diverse features, health-related features, tracks sleep, looks nice or cool, simple smartphone or tablet app, other activities that older adults may be doing, real-time feedback on app or device, smaller design, easy to synchronize, automatic logging of activity, goal tracking, view health information, help section, large and easy-to-press buttons, and easy to see (if falls on the floor)
- Disliked features (in no particular order): looks like a medical device (aesthetics), frequent charging, auto-goal function, inaccuracy, having to wear in bed (if uncomfortable), not capturing all activities, large and rigid band, tethered to the smartphone, uncertainties about water damage and charging, complicated tablet or smartphone, no practical training, does not match clothes, difficult to put on, frequent alarms or notifications, difficult to interact with when on the ankle, not compatible with a smartphone, difficult to handle, and not suited for older adults

The key concepts (order not indicative of prominence or salience) are (1) openness to engage and functional ability of the user; (2) motivation for device use; (3) integration into daily life; and (4) device features.

First-order quotations (raw, primary data, ie, direct participant quotations) and second-order (authors’ interpretations of their primary data) interpretations were used to support the analysis of the *Translation* section. Throughout the *Results* section, first-order quotations (primary study participants) are indicated in italicized quotations and second-order interpretations (primary study authors) are indicated in italics.

#### Openness to Engage and Functional Ability of the User

##### Age-Related Physiology or Comorbidities

Certain age-related characteristics can impact users’ comfort with new technologies, such as hearing loss, limited dexterity, and low vision [13,14]. Older users may be slower to process new information and therefore require simple, visible instructions:

Participants saw that, for senior persons less vigorous than themselves, everyday use of the device could be difficult, cumbersome, and demanding: “It is more difficult for a person less alert than me maybe also using walking aids. It might be tough for them to register like this every day.” [9]

##### Self-efficacy for Technology

In addition to their actual technical skills, an older person’s perceptions of their technical abilities can be a barrier to adoption [13]:

This was reflected by many of our participants in the comments they made about the devices—often relating that they were “not built with us in mind,” that they were created “for someone younger,” and that devices needed a more “tech-savvy” user. [31]

Low self-efficacy for technology can influence users’ attitudes and limit the *perceived ease of use* of a device [13]:

“I was of course a bit worried initially about not being able to handle it. That I would push the wrong button and things like that.” [9]

Insecurities can arise when users encounter usability issues or technical failures and do not have the experience of identifying or resolving the issue:

The participants had felt inexperienced in handling the technical devices and therefore had felt insecure on whether they were doing this correctly. In addition, there were occasions when the technology had not worked properly, and this made the users wonder if the problems experienced were because of incorrect handling. [9]

Individuals with higher self-efficacy for technology are more open to using wearable devices and exploring their features. Users with lower self-efficacy tend to require more support, clear instructions, and additional training to increase their sense of control and prevent device abandonment [13].

##### Exploration and Use of Device Features

Many older adults have a desire to learn more about their health and are interested in various advanced features (which are not always available) [13]. Sometimes, they are frustrated when their device does not have their desired features or when accessing the available features is difficult. Limited technical abilities could hinder the exploration of features and have an effect on behavior change. Clear, simple instructions help users overcome the initial technical hurdles and allow them to explore the features that they desire; this can ultimately lead to continued device use [32].

##### Independence

Users are less open to engaging with a device that is burdensome or limits their independence. A user will not perceive themselves as independent if they have to rely on friends, family, or researchers to help them with device issues. In addition, a device that requires frequent charging will affect the user’s routine and limit the time that they can spend away from a power source [29]. Overall, if a user has to frequently seek assistance with their device, they will not be able to live an independent life with the device, which is often the goal [33]:

“I’m sure it’s there [the support] but it means taking their time, and making my problem their problem. And that’s hard for me to do because of my own attitudes about independence I think. I really resent supervision, which is intrusive and demanding; kinds of stuff like that within the family.” [33]

#### Motivation for Device Use

##### Internal Influences

For many users, a degree of motivation is required to realize behavior change or long-term use. This is not exclusive to activity trackers; some participants do not feel the need to wear a fall detection device, even if they are at risk of falling:

A participant who experienced four falls during the course of the trial explained he did not need the device as, “I don’t consider myself a faller.” [14]

Some users felt that they were too young to need a fall detection device currently:

“You know if were a high fall risk...but at the moment I don’t consider that. When I get old maybe.” [14]

Thus, to successfully incorporate a fall detection device into their life, the person must have a recognized need and personal desire to prioritize their safety.

Activity trackers are often worn to monitor physical activity levels. Some participants were motivated to increase their activity when the device was introduced into their lives:

“I was motivated by the technology, that I freely admit.” [9]

Other participants were already motivated to increase their activities before using the device:

“The technology has no impact on my motivation, I am physically active anyway. I am on the verge of getting diabetes, that is what motivates me the most.” [9]

Both physically active and inactive older adults can lack the motivation to use a wearable device if neither has the desire to change their activity levels. Intrinsic motivation seems to be particularly powerful for users who are inactive but have a strong desire to change this; this group has room to improve, unlike very active people who are already at their desired activity level [13]:

They believed that a wearable device can motivate them to improve their exercise level. This theme was more significant in seniors who did not exercise regularly and seniors with lower income. [13]

Long-term users emphasized the importance of internal motivation (Just do it) where activity trackers were serving as secondary facilitators.... [37]

Those who were already satisfied with their exercise levels saw no benefits from using the device [39]. Equally, those already motivated to exercise felt that the device had no additional effect on their behavior [13].

##### Quantification and Feedback

Some older adults found that quantification of their activity can drive motivation [36,37,39]. Activity tracker users often have a specific goal (eg, increase the daily step count) [36]. Devices that provide feedback help users track their progress [9,36] (eg, “I liked the ability to monitor my progress”).

Each user has a different goal, so the more personalized the feedback, the more effective the device:

Goal setting was perceived important for increasing active behaviour: a quantitative goal was helpful for the user by clarifying if the current activity level was too low. [9]

However, already-active individuals were not always affected by feedback:

“I did not change my exercise habits during the monitoring, I took the same walk as usual in the morning or in the afternoon. It is a goal I have and as a pensioner, I have plenty of time.” [9]

The feedback and features of the device must align with the goals of the user. Some users only need a push (eg, step target). Others have more detailed health monitoring goals (eg, heart rate, sleep, and quantification of multiple activities). People can feel disconnected from a device that does not provide adequate feedback; this can limit a device’s ability to help the user achieve their goals.

The importance of feedback is not limited to activity trackers. Fall detection devices provide feedback in the form of alerts and calls for help. Trial participants said they would like clear feedback about when alerts were activated, who that alert was notifying, and how they could disable the alert [14,29].

##### Awareness of Physical Activity Levels

Using an activity tracker often leads to increased awareness of one’s activity levels, particularly for those who were previously inactive:

“My Fitbit allowed me to personalise my exercise. I learned new things about myself from the fitbit.” [13]

However, increased awareness does not necessarily motivate the user to exercise. The desire to increase exercise levels (before knowing one’s current level) and an achievable exercise goal were more motivating than awareness. Certainly, these devices can show how sedentary users are and remind them to meet their exercise goals but a person must already want to make a lifestyle change:

“It was more informative than motivating, because I had my own agenda that my doctor set out for me to do.” [31]

Thus, activity trackers were more often viewed as a catalyst rather than a creator of behavior change.

##### Emotions Invoked by Device

Feedback on activity can elicit strong emotions among users and can become attached to their results, experiencing positive affect when they meet their goals and negative affect when they do not:

“It was irritating when it is visible that I had been so damn lazy. But it is good to have (the technology).” [36]

Sometimes, users are more concerned with how they connect with a device than the specific output metrics, so that emotional meaning is valued more than actual gains. When a device elicits more positive feelings than negative feelings, users are more inclined to continue use.

Devices can lead to stigma and embarrassment when drawing attention to the public:

“It’s when they don’t say anything you wonder kinda what they’re looking at, cause they do take notice of it.” [14]

False alarms from fall detection devices can lead to disruptions in public [14], and activity alarms from activity trackers can interrupt meetings and social events. Aesthetically, devices with a medical look can lack acceptability, because this can draw extra attention and many older adults do not want to be viewed as a “patient”:

“...what I was wearing was sheer, and would show this light which everybody was curious about, and it just didn’t look good with, I didn’t want to wear it.” [14]

##### Extrinsic Motivation—Social Support

For many users, the social network around the device is key to its continued use [13,36]. Social interaction and engagement and peer group attitudes toward technology are key factors influencing adoption. Ongoing peer support and encouragement can positively influence adherence:

“Meeting with others in the sense of: did they experience the same thing? Do they need encouragement? Can something they’re doing encourage me to alter behaviours?” [36]

Peers can also help troubleshoot issues and provide hints for maximizing user benefits [36]. Social support is important for long-term use, because intrinsic motivation can waver over time. The device can also act as its own “social support” if it provides good feedback and is easy to interact with. This is important for older adults who are isolated (physically or socially) and therefore might need help to establish a support group:

Long-term users indicated social support to be the main motivational factor, with the focus on building relationships around daily activity routines. Long-term users were better prepared to modify the social environment around them to maintain an active lifestyle, receive positive feedback, and seek accountability from others. [37]

##### Promotion by Health Care Staff

For those using a device for medical purposes, the input and encouragement of a health professional can be important for adoption and continued use. Learning about the device from professionals can help overcome barriers to adoption and ultimately meet their goals:

“But if someone can guide you through it, I think any of them, once you start using them you would probably use it. But I wouldn’t go to Best Buy I wouldn’t have thought to go to best buy. If it’s for my health, I would think to go to a pharmacy.” [31]

#### Integration Into Daily Life

To be successfully integrated into the user’s life, the device must not only be acceptable and reliable, it must also be perceived by the user to add value to their life. The ease of integration is often determined by the purpose and features of a device and the reliability of the device’s functions. Certain design features, such as appearance, weight, material, dimensions, and comfort, are particularly important. If the wearable device mimics a device already in the user’s life (eg, wristwatch), it can be seamlessly integrated:

“Then, it becomes a habit. And this is precisely what happened to me with my watch [reference to the AT].” [44]

If the device does not have the user’s desired features (eg, swimming, activity history, or GPS tracking), the user’s perceived value of the device may be low:

“Really, after the bloom got off the rose, I didn’t like anything about it.” [34]

Conversely, users may tolerate design faults if they value the device.

A common barrier to acceptance is its unreliability. When someone cannot rely on a device to serve its purpose or give accurate feedback, it loses value, and the motivation to wear the device can wane. This is evident in the authors’ conclusions:

Some participants...questioned whether the result was correct. This reduced their motivation for being monitored. [9]

It is also evident in the raw participant data:

“...We began to think that it wasn’t accurate, so it lost its appeal.” [34]

Critically, the device should not negatively affect the user’s routine. Frequent charging, not being waterproof, being tethered to a smartphone, and being difficult to put on and take off are examples of features that can disrupt a user’s routine [13,14]. When this happens, especially for older users, the device does not integrate into the user’s life and loses its value.

#### Device Features

Participants across the 20 included studies generally preferred devices that have the following features: waterproof, small in size, comfortable (especially if worn at night), aesthetically pleasing (fashionable; not like a medical device), with an easy-to-read display, a long battery life, and a thin, flexible band (if worn on the wrist). They enjoyed using device features that counted their steps, tracked their location using GPS, automatically logged their activity, measured health parameters (heart rate, blood pressure, or sleep), updated them on activity goals, automatically contacted help in the event of a fall, and synchronized automatically with their other devices. They like devices that are easy to attach, are secure, do not interfere with clothing, and are easy to handle.

The participants disliked devices that were inaccurate, required frequent charging, were uncomfortable, tethered to a smartphone, were difficult to attach, were not compatible with their smartphone, were not suited for older users, or do not capture all of their daily activities. They especially disliked devices without adequate instructions to help them troubleshoot issues or turn off annoying alarms.

### Synthesis

#### Summary of Synthesis Process

The first-order (quotations), second-order (individual themes extracted from each paper), and third-order (key concept) interpretations were considered as a whole to develop a line-of-argument synthesis.

The experience of integrating a device into everyday life is a dynamic process of assessing the added value of the device and is influenced by a range of interrelated intrinsic (internal motivation, functional ability, interest, and openness) and extrinsic (external motivation, training, device characteristics, functionality, and feedback) factors. Many factors influence whether an older adult sees a device as worth wearing, and the appraisal and balance of these factors tell the user whether the device adds value to their life.

#### Line of Argument

**Figure 2:**
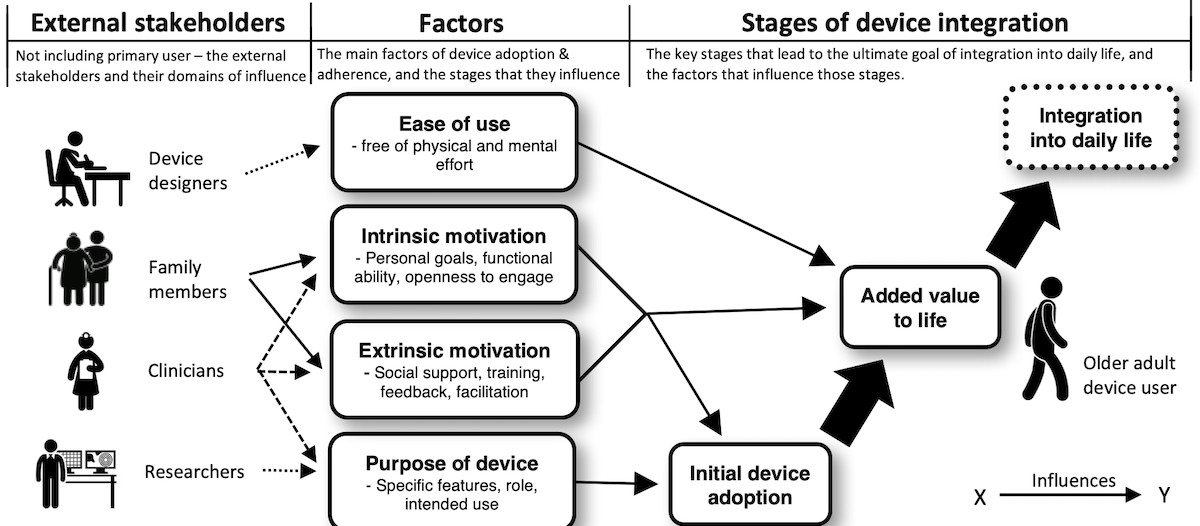
Conceptual model developed from the line-of-argument synthesis.

We developed a line of argument to describe the factors that influence successful integration (Figure 2). Our line of argument takes the form of a “conceptual model” of the factors that lead to the integration of the device into the user’s life. Our conceptual model describes how motivation, ease of use, and device purpose determine whether a device will add value to the user’s life, which subsequently determines if the device will be integrated into their life.

User motivation is key. Without motivation (eg, symptom monitoring), the user will view the device as just another piece of technology. On the basis of the data collated in this synthesis, we found that older adults do not adopt new technologies because of their novelty. We found that motivation comes in two forms: intrinsic and extrinsic motivation. They influence both the user’s initial reason to adopt a device and to sustain its use.

Intrinsic motivation describes a user’s personal connection to a device. Initially, the user must be motivated to make a change in their life that will be supported by adopting a wearable device (eg, increasing physical activity or detecting falls). Intrinsic motivation is often required for individuals to adopt a device initially. The device itself does not create motivation; many users commented that while being able to view their daily step count is interesting, it does not spur them to change their physical activity habits unless they are already motivated to do so. Intrinsic motivation is also important for a device to add long-term value to a user’s life. It is necessary to overcome some of the usability hurdles that users face when they adopt a device. It also fuels continued use as the initial novelty wears off.

Extrinsic motivation is another important contributor to device adoption and added value. This includes factors such as training, technical support, promotion, support from health professionals, peer support, and device feedback. Initially, extrinsic motivation influences device adoption through the practicalities of acquiring and setting up the device and learning how to use its features. Older adults are often asked to adopt a device for fall detection or as part of a treatment or health regime. This form of extrinsic motivation often leads to device adoption but may not contribute to added value if other extrinsic factors (eg, technical and peer support) are not present. Technical support was frequently cited as a crucial extrinsic motivator, both initially and over time. Good technical support connotes added value because it gives the user the confidence to explore the device’s features and supports integration into the user’s life. Peer support is another important extrinsic motivation factor that contributes to both device adoption and added value. It comes in many forms and is unique for each user, but its importance is universal. Social support encourages older adults to adopt wearable devices and motivates continued use. Conversely, reliance on social support (eg, having to bother someone for assistance) limits the user’s independence and could be a barrier to continued use. Together, the factors of intrinsic and extrinsic motivation influence whether an individual will adopt a device and whether the device will continue to add value to their life.

The purpose of the device (fall detection, step count, etc) is the main reason why older adults adopt it, and it is what initially draws a user to that specific device. Unlike those of a younger generation, older adults do not tend to use new technology simply because they have fun features. They view devices as tools and expect them to serve their purposes with accuracy and reliability. The purpose of a device (and its features) is key to adoption. Older adults are unlikely to adopt a device that does not fulfill pre-existing needs. The purpose of the device also adds value and facilitates integration as the user expands their relationship with the device. Upon adoption, the user evaluates whether, and to what extent, the device serves its intended purpose. A device that continues to serve its intended purpose (or serves additional purposes as the user becomes more familiar with its features) is perceived to add value, leading to integration as the user relies on that device to fulfill an important need in their life.

Along with motivation and purpose, ease of use also predicts added value. This is defined as the degree to which a device is free of physical and mental effort for users. Specific device features (eg, battery life and touch screen menus) influence ease of use, as do general features such as access to simple instructions and the amount of interaction required. An easy-to-use device adds value by reducing the burden of using the device. This allows users to focus on their motivators and the fundamental purpose of the device.

Added value to life is the ultimate contributor to successful integration into daily life. The added value is the resulting balance of motivators (or lack thereof), device features (and their accuracy), ease of use, device purpose, and user experience. When the negatives outweigh the positives, the device will most likely not be integrated into everyday life.

## Discussion

### Principal Findings

This is the first study to systematically review and synthesize the qualitative literature on older adults’ experiences with wearable devices. This meta-synthesis collated the experiences of 349 trial participants and presented the key factors that influence user acceptance and adherence. These factors include intrinsic and extrinsic motivation to use the device; the purpose of the device and how it relates to the user’s expectations and needs; and the ease of use and functional ability of the user. The user’s appraisal of these factors determines the level of value added by the device to the life of each user.

Motivation for device use comes in two forms (intrinsic and extrinsic) and encompasses many aspects of the user experience. According to our line-of-argument synthesis, motivation influences both device adoption and added value. Motivation seems to be as, if not more, important for older adults than the actual device features. Moreover, the user’s needs and the support structure around the device—aspects that are often overlooked—seem to play a crucial role in long-term adoption.

### Comparisons With Previous Work

Our bottom-up inductive qualitative synthesis supports the findings of existing theory-bound models of technology acceptance. It was not intended from the outset that our conceptual model would tie in with quantitative models such as the Technology Acceptance Model (TAM) and the value-based adoption model (VAM). We felt that the TAM and VAM could be used to structure and contextualize our findings. These models use quantitative surveys to test hypotheses about factors that predict the intention to use. For example, the TAM shows that perceived ease of use, perceived usefulness, and attitude toward the system predict intention to use [51]. The development of these models does not involve trial components or qualitative methods. Therefore, our study should not be directly compared with the TAM or any other acceptance model and should instead provide inspiration for hypotheses to test in future iterations of wearable device acceptance models.

Originally designed to describe the acceptance of information services in organizations [51], the TAM has only recently been applied to wearable devices [52-55]. In contrast to the TAM, the authors of the VAM recognized that most consumers adopt mobile technologies for personal purposes and that the cost of voluntary adoption is borne by the individual, not the organization [56]. According to the VAM, perceived sacrifices (cost and technicality) seem to have a greater impact than perceived benefits (usefulness and enjoyment) on perceived value. Many participants in our review alluded to this balance between sacrifices and benefits. They described how a device that disrupts their routine or limits their independence is not worth the hassle, especially if the features (eg, counting steps) are not beneficial to them.

The TAM and VAM inspired the recent development of a smart wearables acceptance model for older adults by Li et al [57]. Along with established acceptance factors such as perceived usefulness [51], Li et al [57] included older adult–specific factors such as self-reported health conditions, perceived social risk, performance risk, and social influence. Their results supported their hypothesis that facilitating conditions positively predicted intention to use, a finding supported by our study. They also showed that the self-reported health status is a negative predictor of use, suggesting that older adults with a better health status are not likely to require these technologies. Our results show a similar trend; older adults are motivated to use a device if they have a need (eg, monitor symptoms and fall risk). In contrast to our findings, 95.9% (140/146) of their participants perceived minimal or no social risk when using a wearable device. This may be because their participants were not offered the opportunity to wear the device in public. Several of the participants in our review (who did wear their devices in public) described feelings of embarrassment or stigma when the device intrudes on the user’s life.

Our review points to age-related factors that can influence acceptance, such as experience with technology and openness to engage. A systematic review of factors influencing acceptance of technology for ageing-in-place found a similar phenomenon and discussed the effect of age and chronic illness on the acceptance of vital sign monitoring systems [58,59]. The systematic review also highlighted the impact of social support from family, friends, professional caregivers, and peers [58]. In their 2014 review of determinants and barriers, Lee and Coughlin [60] described eight similar factors (value, usability, technical support, social support, emotions, independence, experience, and confidence) and two additional factors that our review did not uncover (affordability and accessibility) [60]. Similar findings from these studies indicate a convergence of the field toward an understanding of the key factors that influence adoption and adherence.

### Relevance for Researchers, Clinicians, and Designers

When designing future wearable device acceptance models for older adults, researchers should consider the multiple stages of device use that follow the initial “intention to use.” Furthermore, in the user experience, concepts such as added value become relevant, which may have a different set of predictors than the initial intention to use. In the development of the Senior Technology Acceptance and Adoption Model (STAM), Renaud and van Biljon [61] related certain acceptance factors to adoption stages. The STAM includes factors such as *confirmed usefulness* and *ease of learning and use*. Using qualitative methods to develop their model, Renaud and van Biljon [61] used these factors to explain why older adults do not reach the final adoption phase and never fully accept technology. Yu-Huei [62] adapted the STAM to wearable devices and added two additional factors, information source and group behavior, which emerged from their qualitative analysis of older adults in Taiwan.

Our study proposed several predictors that could be tested as a part of future model development studies. First, motivation is key and seems to be a constant driving force throughout the user experience. Researchers should question users on both intrinsic and extrinsic motivators to see if these factors predict integration. Second, the purpose of the device (and whether the user aligns with that purpose) should be investigated as a predictor of device adoption and added value. Finally, ease of use should be considered within the context of older adults, as done by Yu-Huei [62]. The technical abilities of older adults may differ from those of younger generations, and certain medical conditions may hinder functional abilities.

Researchers play a role in validating (or refuting) the findings of this review. Older adults require a specific wearable device acceptance model because they are a distinct population from the individuals used to develop the TAM and other wearable device acceptance models [54]. As opposed to smartphones and embodied conversational agents, wearable devices are designed to be used individually and continuously, which leads to a unique set of influencing factors.

For clinical trial researchers, we stress the need to provide extrinsic motivation for their participants by conveying the importance of the device. They should also provide training and technical support to facilitate ease of use. Clinicians using wearable devices in their private practice can provide extrinsic motivation by clearly explaining the device’s purpose and the meaning of measurements to their patients. They should also provide encouragement and technical support. They can support intrinsic motivation by learning about their patients’ health goals and desires to use a wearable device.

Although not explicitly stated, several of the included studies used a user-centered design approach [9,14,29]. A user-centered design, though often overlooked, is essential in the development of wearable devices, particularly if the designers are not a part of the intended demographic. Designers, clinicians, and researchers can collaborate with older adults to address all aspects of wearable device use. As the studies in our review demonstrate, trials and qualitative data are valuable tools for designers using the user-centered design approach to explore the long-term use of their products. Some device features become relevant only after a period of usage. For the benefit of designers, we summarized some of the features that were commonly discussed in our review. The study participants discussed many design features that are specific to the devices in question, and thus features could not be compared between the studies. However, we can make a few generalizations. First, designers should not take for granted that older adults will accept every design feature. Second, a device should serve the purpose, and the primary function of the device should be reliable and easy to use; some older adults are interested in advanced features, but not all are. Third, participants disliked unnecessary interactions with the device, such as frequent charging, responding to alerts, and maintaining a Bluetooth connection to their smartphone. An ideal device requires little maintenance and only requires interaction to monitor the data and obtain device feedback. Finally, a wearable device should be easy to take on and off, comfortable to wear at night, and waterproof. It should cause minimal disruption in users’ lives, be aesthetically pleasing, and should not draw attention to the user or single them out as a patient; it should not interfere with clothing; and it should have a silent mode to prevent unnecessary disruptions.

### Strengths, Limitations, and Future Directions

Measures such as practicing reflexivity and using 2 reviewers maximized the quality of this meta-synthesis. The authors are an experienced multidisciplinary team (geriatric medicine, psychology, epidemiology, and engineering) with expertise in qualitative approaches. By following the eMERGe reporting guidance (see Table S1 in Multimedia Appendix 2 [50]), we communicated our methodology with transparency, including our study’s limitations. Although both reviewers (KM and LK) collaborated to generate the search strategy and inclusion criteria, because the second reviewer only screened a sample of the references, some relevant studies may have been excluded during the screening process. Our database search only included 70% (14/20) of the included studies. The additional 6 studies were located by manually searching the reference lists and searching Google Scholar, highlighting the limitations of our search strategy. For example, we should have included the search term “senior*” to find studies such as the Abouzahra and Ghasemaghaei [13] study. It is worth noting that this issue is more common in qualitative reviews than in quantitative reviews because of the poor and inconsistent indexing of qualitative research in databases [63]. While we searched four databases, searching for additional databases (eg, Scopus and ISI Web of Science) would have strengthened our study.

The content of our results and the line-of-argument conceptual model were contingent on the data collected from the broad inclusion criteria. This is both a strength and a weakness of qualitative syntheses; it affords reviewers the flexibility to uncover new ideas but it dictates which questions can be answered. Some studies lacked rich descriptions and interpretations of their findings or did not provide sufficient context (about the sample, the device, or the procedures). This limited the contributions of some studies to the meta-synthesis [64], regardless of their ETQS quality score. Studies with low ETQS scores often fell short of simple aspects, such as not reporting the location of the study. Ultimately, the results of this study are based on the synthesis of qualitative data, which is inherently subjective. Our line-of-argument and conceptual model provide suggestions, but the full development of a wearable device acceptance model for older adults will take place with a more rigorous study design.

All studies included in this review were published in English and conducted in Western countries. The findings may not represent countries with different cultures, access to wearables, and income levels. Several studies included short trial periods and a few participants. Each study evaluated a different device (or set of devices), which restricted comparisons between studies. Future research would benefit from long-term trials using in-depth qualitative methods to evaluate the drivers of acceptance and adherence. Future research must also include the views of older adults who use wearable devices as part of clinical care, not just a research trial. An alternative set of predictors might be relevant to participants who use a device for a specific health purpose.

### Conclusions

This review found that several key factors influence the acceptance and use of wearable devices by older adults. These include intrinsic and extrinsic motivation for device use, ease of use, device purpose, and perceived added value to the user’s life. Designers, clinicians, and researchers should be aware that useful device features alone do not lead to continued use. To overcome the usability barriers (eg, limited technical ability), an older adult must be motivated to use a device because it serves a useful purpose. A support structure should be placed around the user that fosters motivation, encourages engagement with peers, and adapts to the user’s preferences. Future research should evaluate our conceptual model by validating our proposed predictors and conducting long-term wearable device trials that use qualitative methods to comprehensively address the multiple stages of device use and the many factors that contribute to adherence.

## Appendix

Multimedia Appendix 1 Evaluation tool for the qualitative studies quality appraisal checklist.

Multimedia Appendix 2 The Meta-Ethnography Reporting Guidance checklist.

## Abbreviations

ETQS: Evaluation Tool for Qualitative Studies
SENDoc: Smart Sensor Devices for Rehabilitation and Connected Healthy
eMERGe: Meta-Ethnography Reporting Guidance
STAM: Senior Technology Acceptance and Adoption Model
TAM: Technology Acceptance Model
VAM: value-based adoption model
