# Supplementary material for: Older Adults’ Experiences With Using Wearable Devices: Qualitative Systematic Review and Meta-synthesis
Source: JMIR Mhealth Uhealth. 2021 Jun 3;9(6):e23832. doi: 10.2196/23832 (PMC8212622; doi:10.2196/23832)
Supplement: Multimedia Appendix 1 [file mhealth_v9i6e23832_app1.docx]

| **First author, year** | **Phenomena under study** | **Theoretical framework** | **Setting** | **Sampling** | **Depth of Perspective** | **Ethics** | **Data Collection** | **Data Analysis** | **Positionality/ reflexivity** | **Policy/ Practice Implications** | **Quality Score** |
| --- | --- | --- | --- | --- | --- | --- | --- | --- | --- | --- | --- |
| Abouzahra, 2019 | **√** | **X** | **X** | **X** | **√** | **√** | **√** | **√** | **X** | **√** | 6 |
| Batsis, 2016 | **X** | **X** | **√** | **√** | **√** | **X** | **X** | **√** | **X** | **√** | 5 |
| Demiris, 2015 | **√** | **√** | **√** | **√** | **√** | **X** | **√** | **√** | **X** | **√** | 8 |
| Ehn, 2015 | **√** | **√** | **√** | **√** | **√** | **√** | **√** | **√** | **√** | **√** | 10 |
| Farina, 2019 | **√** | **√** | **X** | **√** | **√** | **√** | **√** | **√** | **X** | **√** | 8 |
| Fausset, 2018 | **√** | **√** | **√** | **√** | **X** | **X** | **√** | **X** | **X** | **X** | 5 |
| Flogel, 2018 | **X** | **X** | **√** | **√** | **√** | **√** | **√** | **√** | **X** | **√** | 7 |
| Hermanns, 2019 | **√** | **√** | **√** | **√** | **√** | **√** | **X** | **√** | **X** | **√** | 8 |
| Kononova, 2019 | **√** | **√** | **√** | **√** | **√** | **√** | **√** | **√** | **X** | **√** | 9 |
| Lee, 2019 | **√** | **√** | **√** | **√** | **√** | **√** | **√** | **X** | **X** | **√** | 8 |
| Mercer, 2016 | **√** | **√** | **X** | **√** | **√** | **√** | **√** | **√** | **X** | **√** | 8 |
| Nguyen, 2016 | **√** | **√** | **X** | **√** | **√** | **√** | **√** | **√** | **X** | **√** | 8 |
| Preusse, 2016 | **√** | **√** | **X** | **√** | **X** | **√** | **√** | **√** | **X** | **√** | 7 |
| Puri, 2017 | **√** | **√** | **X** | **√** | **√** | **√** | **√** | **√** | **√** | **√** | 9 |
| Rosales, 2018 | **√** | **X** | **√** | **√** | **X** | **√** | **√** | **√** | **X** | **X** | 6 |
| Schlomann, 2016 | **√** | **√** | **X** | **√** | **X** | **X** | **√** | **√** | **X** | **√** | 6 |
| Schlomann, 2017 | **√** | **√** | **X** | **X** | **X** | **√** | **√** | **√** | **X** | **√** | 6 |
| Thilo, 2019 | **√** | **√** | **√** | **√** | **X** | **√** | **√** | **√** | **X** | **√** | 8 |
| Thorpe, 2019 | **√** | **√** | **√** | **√** | **X** | **X** | **√** | **X** | **X** | **√** | 6 |
| Zhou, 2018 | **√** | **√** | **X** | **X** | **X** | **√** | **√** | **√** | **X** | **√** | 6 |
