# Supplementary material for: Older Adults’ Experiences With Using Wearable Devices: Qualitative Systematic Review and Meta-synthesis
Source: JMIR Mhealth Uhealth. 2021 Jun 3;9(6):e23832. doi: 10.2196/23832 (PMC8212622; doi:10.2196/23832)
Supplement: Multimedia Appendix 2 [file mhealth_v9i6e23832_app2.docx]

Table 3: the eMERGe reporting guidance checklist

| No. | Criteria | Description | Reported |
| --- | --- | --- | --- |
| Phase 1⎯Selecting meta-ethnography and getting started | | |  |
| 1 | Rationale and context for the meta-ethnography | Describe the gap in research or knowledge to be filled by the meta-ethnography, and the wider context of the meta-ethnography | **√** |
| 2 | Aim(s) of the meta-ethnography | Describe the meta-ethnography aim(s) | **√** |
| 3 | Focus of the meta-ethnography | Describe the meta-ethnography review question(s) (or objectives) | **√** |
| 4 | Rationale for using meta-ethnography | Explain why meta-ethnography was considered the most appropriate qualitative synthesis methodology | **√** |
| Phase 2⎯Deciding what is relevant | | |  |
| 5 | Search strategy | Describe the rationale for the literature search strategy | **√** |
| 6 | Search process | Describe how the literature searching was carried out and by whom | **√** |
| 7 | Selecting primary studies | Describe the process of study screening and selection, and who was involved | **√** |
| 8 | Outcome of study selection | Describe the results of study searches and screening | **√** |
| Phase 3⎯Reading the included studies | | |  |
| 9 | Reading and data extraction process | Describe the reading and data extraction method and processes | **√** |
| 10 | Presenting characteristics of included studies | Describe characteristics of the included studies | **√** |
| Phase 4⎯Determining how studies are related | | |  |
| 11 | Process for determining how studies are related | Describe the methods and processes for determining how the included studies are related  -Which aspects of studies were compared  AND  -How the studies were compared | **√** |
| 12 | Outcome of relating studies | Describe how studies relate to each other | **√** |
| Phase 5⎯Translating studies into one another | | |  |
| 13 | Process of translating studies | Describe the methods of translation:  - Describe steps taken to preserve the  context and meaning of the relationships between concepts within and across studies  - Describe how the reciprocal and refutational translations were conducted  - Describe how potential alternative interpretations or explanations were considered in the translations | **√** |
| 14 | Outcome of translation | Describe the interpretive findings of the translation | **√** |
| Phase 6⎯Synthesizing translations | | |  |
| 15 | Synthesis process | Describe the methods used to develop overarching concepts (“synthesised translations”) Describe how potential alternative interpretations or explanations were considered in the synthesis | **√** |
| 16 | Outcome of synthesis process | Describe the new theory, conceptual framework, model, configuration, or interpretation of data developed from the synthesis | **√** |
| Phase 7⎯Expressing the synthesis | | |  |
| 17 | Summary of findings | Summarize the main interpretive findings of the translation and synthesis and compare them to existing literature | **√** |
| 18 | Strengths, limitations, and reflexivity | Reflect on and describe the strengths and limitations of the synthesis: - Methodological aspects—for example, describe how the synthesis findings were influenced by the nature of the included studies and how the meta-ethnography was conducted.  - Reflexivity—for example, the impact of the research team on the synthesis findings | **√** |
| 19 | Recommendations and conclusions | Describe the implications of the synthesis | **√** |
